# Bis-choline tetrathiomolybdate prevents copper-induced blood–brain barrier damage

**DOI:** 10.26508/lsa.202101164

**Published:** 2021-12-02

**Authors:** Sabine Borchard, Stefanie Raschke, Krzysztof M Zak, Carola Eberhagen, Claudia Einer, Elisabeth Weber, Sandra M Müller, Bernhard Michalke, Josef Lichtmannegger, Albrecht Wieser, Tamara Rieder, Grzegorz M Popowicz, Jerzy Adamski, Martin Klingenspor, Andrew H Coles, Ruth Viana, Mikkel H Vendelbo, Thomas D Sandahl, Tanja Schwerdtle, Thomas Plitz, Hans Zischka

**Affiliations:** 1 Institute of Molecular Toxicology and Pharmacology, Helmholtz Center Munich, German Research Center for Environmental Health, Neuherberg, Germany; 2 Institute of Nutritional Science, University of Potsdam, Nuthetal, Germany; 3 TraceAge–Deutsche Forschungsgemeinschaft Research Unit on Interactions of Essential Trace Elements in Healthy and Diseased Elderly (Forschungsgruppe 2558), Berlin-Potsdam-Jena-Wuppertal, Germany; 4 Institute of Structural Biology, Helmholtz Center Munich, German Research Center for Environmental Health, Neuherberg, Germany; 5 Research Unit Analytical BioGeoChemistry, Helmholtz Center Munich, German Research Center for Environmental Health, Neuherberg, Germany; 6 Institute of Radiation Medicine, Helmholtz Center Munich, German Research Center for Environmental Health, Neuherberg, Germany; 7 Technical University Munich, School of Medicine, Institute of Toxicology and Environmental Hygiene, Munich, Germany; 8 Research Unit Molecular Endocrinology and Metabolism, Genome Analysis Center, Helmholtz Zentrum München, German Research Center for Environmental Health, Neuherberg, Germany; 9 Lehrstuhl für Experimentelle Genetik, Technical University Munich, Freising-Weihenstephan, Germany; 10 Department of Biochemistry, Yong Loo Lin School of Medicine, National University of Singapore, Singapore, Singapore; 11 Chair of Molecular Nutritional Medicine, Technical University of Munich, School of Life Sciences Weihenstephan, Freising, Germany; 12 Else-Kröner Fresenius Center for Nutritional Medicine, Technical University of Munich, Freising, Germany; 13 Alexion AstraZeneca Rare Disease, Boston, MA, USA; 14 Department of Nuclear Medicine and Positron Emission Tomography Centre, Aarhus University Hospital, Aarhus, Denmark; 15 Department of Biomedicine, Aarhus University, Aarhus C, Denmark; 16 Medical Department Hepatology and Gastroenterology, Aarhus University Hospital, Aarhus, Denmark; 17 Wilson Therapeutics AB, Stockholm, Sweden

## Abstract

In Wilson disease, excessive copper accumulates in patients’ livers and may, upon serum leakage, severely affect the brain according to current viewpoints. Present remedies aim at avoiding copper toxicity by chelation, for example, by D-penicillamine (DPA) or bis-choline tetrathiomolybdate (ALXN1840), the latter with a very high copper affinity. Hence, ALXN1840 may potentially avoid neurological deterioration that frequently occurs upon DPA treatment. As the etiology of such worsening is unclear, we reasoned that copper loosely bound to albumin, that is, mimicking a potential liver copper leakage into blood, may damage cells that constitute the blood-brain barrier, which was found to be the case in an *in vitro* model using primary porcine brain capillary endothelial cells. Such blood–brain barrier damage was avoided by ALXN1840, plausibly due to firm protein embedding of the chelator bound copper, but not by DPA. Mitochondrial protection was observed, a prerequisite for blood–brain barrier integrity. Thus, high-affinity copper chelators may minimize such deterioration in the treatment of neurologic Wilson disease.

## Abstract (precis)

The blood–brain barrier endothelial cell monolayer becomes permeable to elevated copper loosely bound to albumin, which can be avoided by a high-affinity copper chelator but not by D-penicillamine.

## Introduction

In 1912, Samuel Alexander Kinnier Wilson reported a fatal neurological disease characterized by a progressive degeneration of the lenticular nucleus that additionally was associated with liver cirrhosis (1). Today, we know that Wilson disease (WD) is due to an impairment of the mostly liver-residing copper-transporting ATPase ATP7B (2, 3, 4). ATP7B defects cause massive liver copper accumulation and current viewpoints state that this copper may leak into the circulation (5). In WD, patients’ blood copper is not tightly incorporated into the copper-bearing plasma protein ceruloplasmin, but potentially available for its accumulation in peripheral organs, especially the brain (6). Indeed, a correlation between a progressively elevated serum concentration of non-ceruloplasmin copper (NCC) and the neurological severity has been described (7). Moreover, in the WD animal model toxic milk mouse, there is some experimental evidence for this route. These mice appear with enormous liver copper accumulations, whereas modest elevations are seen in the spleen, kidney, and brain (8). Upon intragastric D-penicillamine (DPA) administration, within days, a significant increase in copper in the serum and also in the brain was demonstrated in these mice (9), and thus one may conclude that it is the DPA taken up by the portal vein that liberates liver copper to cause serum and brain copper elevations.

In WD patients, upon years or even decades of accumulation, brain copper concentrations may reach up to 450 μg/g dry weight (versus 7–60 μg/g dry weight in controls) (10, 11, 12), considered to be the prime toxic condition that causes brain lesions and neurologic symptoms (e.g., dysarthria and parkinsonism) (13). Nevertheless, many aspects of the pathophysiology of neurologic WD are still rather circumstantial, lack clinical evidence, or are unknown. Among them is the issue how elevated NCC enters and accumulates in the brain.

In WD patients, blood copper is mainly bound to albumin and amino acids (14, 15, 16). Being the most abundant plasma protein (35–50 g/l; 500–750 μM), albumin has a huge copper binding capacity and may bind up to five copper ions at pH 7.4 (17). A first copper ion is tightly bound to the N terminus (K_d_ = 0.9 × 10^−12^ M (18)–6.7 × 10^−17^ M (19)), a second one to a multi-metal binding site with intermediate affinity (K_d_ = 1.91 × 10^−7^ M (17)), and the remaining three copper ions are relatively loosely attached to presently uncharacterized sites (K_d_ = 6.25 × 10^−6^ M (17)). In this respect, the amino acid histidine may play a minor role because of its comparatively lower plasma concentration (≈100 μM (15)) and intermediate copper affinity (K_d_ ≈ 10^−9 ^(20)). Thus, in plasma, several binding partners for copper exist with either high capacity and/or high affinity. In WD patients, total blood copper concentrations of 0.5–16.6 μM have been observed (21, 22), among them a relatively low concentration of 1–5 μM copper that may be exchangeable as determined by EDTA (K_d_ = 1.26 × 10^−16^ M (18)) chelation experiments (23). This raises the issue of how excess brain copper accumulation may occur. One mechanism may be a constant competition for and uptake of copper into the brain via the high-affinity transporter CTR1 (K_d_ ≈ 10^−14^ M), possibly linked to an impaired copper re-export into the blood due to ATP7B defects (24). CTR1 and ATP7B are present at the blood-facing membrane of endothelial cells that form the blood–brain barrier (BBB) along with astrocytes and pericytes (25). Such competition at relatively low NCC blood copper, together with a one-way copper route into the brain due to ATP7B absence, may explain the observed long clinical silence, sometime lasting decades, of neurologic complications in WD patients. Another, not mutually exclusive possibility is that periods of copper-induced liver damage may cause intense “blood copper pulses,” thereby causing brain copper accumulation and damage. In fact, clinically relevant fluctuations in neurologic symptoms, sometimes multiple times per day, with varying degrees of severity have been reported. These symptoms may be exacerbated by stress, concurrent illnesses, or medications (26). Such brain damage of NCC may start at the BBB that then may facilitate further unregulated copper entry into the brain. In agreement with this hypothesis, Stuerenburg described disturbances of the BBB in neurologic WD patients, as indicated by an increased ratio of albumin presence in cerebrospinal fluid versus serum (27). Moreover, copper chelation treatments that bind excess liver copper, may secondarily cause such copper pulses. Here, as has been demonstrated in toxic milk mice, the chelator DPA directed copper to the blood, causing elevations in brain (9). Indeed, treatment of neurologic WD patients with DPA can lead to dramatic symptom worsening as reported in 19–52% of the patients (28, 29, 30, 31, 32, 33, 34), especially shortly upon treatment onset. Such neurological worsening is not typically reported in tetrathiomolybdate (TTM)-treated patients (35, 36, 37). As DPA has a lower copper affinity (K_d_ = 2.4 × 10^−16^ M (38)) than TTM (K_d_ = 2.3 × 10^−20^ M (38)), because of its tight binding, competition for copper in the blood may be diminished by the latter, possibly leading to lower BBB and/or brain damage.

To shed light into these hypotheses, we have studied the dose-dependent copper-induced damage to the constituting cells of the BBB using increasing copper amounts bound to albumin, that is, from tightly to more loosely albumin-bound copper, thereby mimicking hypothetical blood copper pulses. Importantly, we find that such damage can be avoided upon presence of the high-affinity chelator bis-choline TTM (ALXN1840), but not by DPA.

## Results

### Copper chelators elevate blood copper differentially

**Figure 1.**
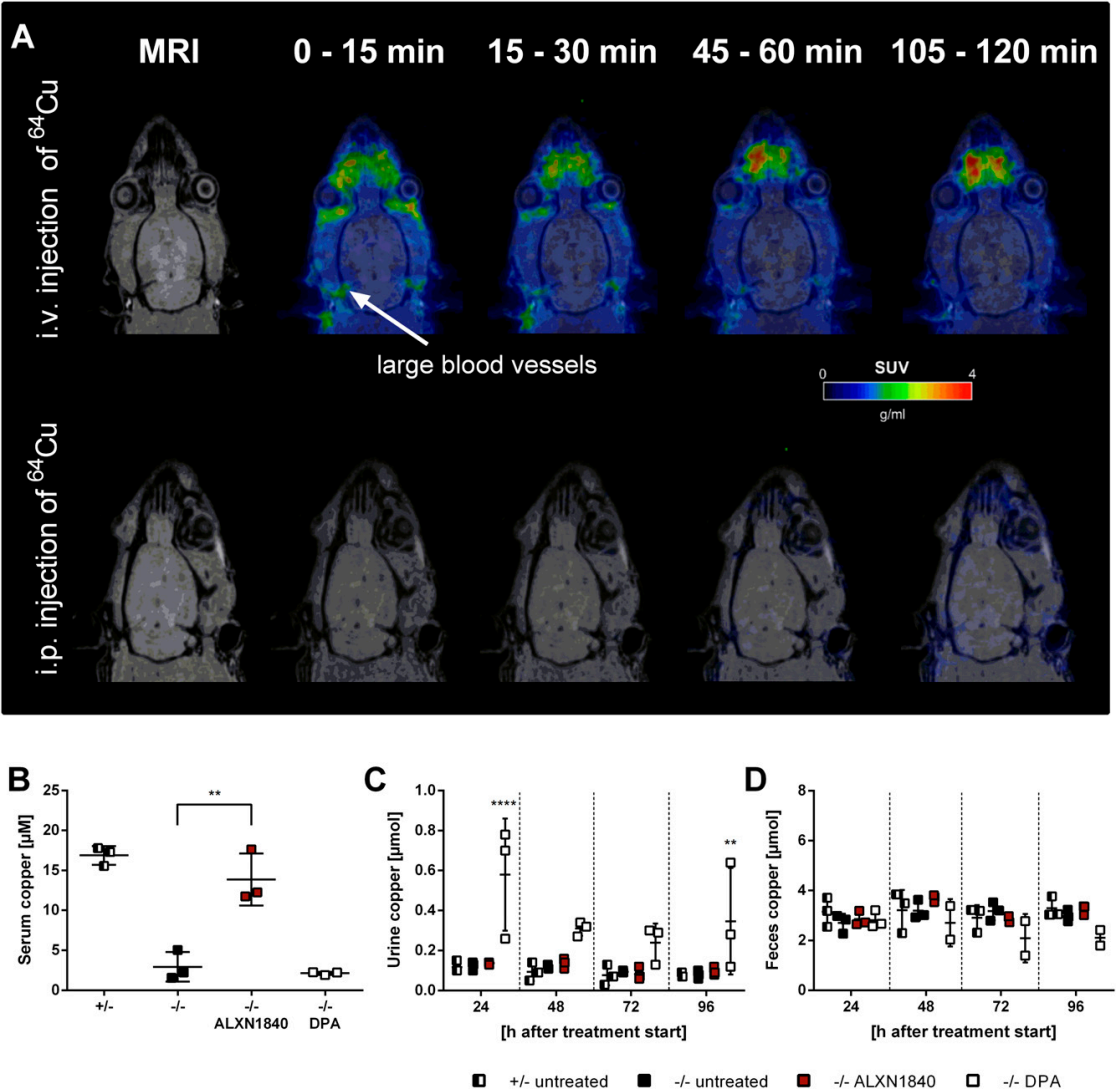
ALXN1840 and DPA increase blood copper levels. **(A)** Positron emission tomography scan of wild-type rats with ^64^Cu injected either i.v. or i.p. I.v. injection results in a fast and high ^64^Cu signal in brain proximate vessels in contrast to i.p.–injected rats. **(B)** Significantly increased serum copper levels are detected in *Atp7b*^*−/−*^ rats treated with ALXN1840 (for 4 d) upon euthanasia, in contrast to DPA treatment (N = 3). **(C)** During DPA treatment of *Atp7b*^*−/−*^ rats, a significantly increased urinary copper excretion is detected (N = 3). **(D)** No increased fecal copper excretion is observed during ALXN1840 and DPA treatments (N = 3). One-way ANOVA with Dunnett’s multiple comparisons test was used for statistical analysis. *P < 0.05, **P < 0.01, ***P < 0.001, ****P < 0.0001.

**Figure S1.**
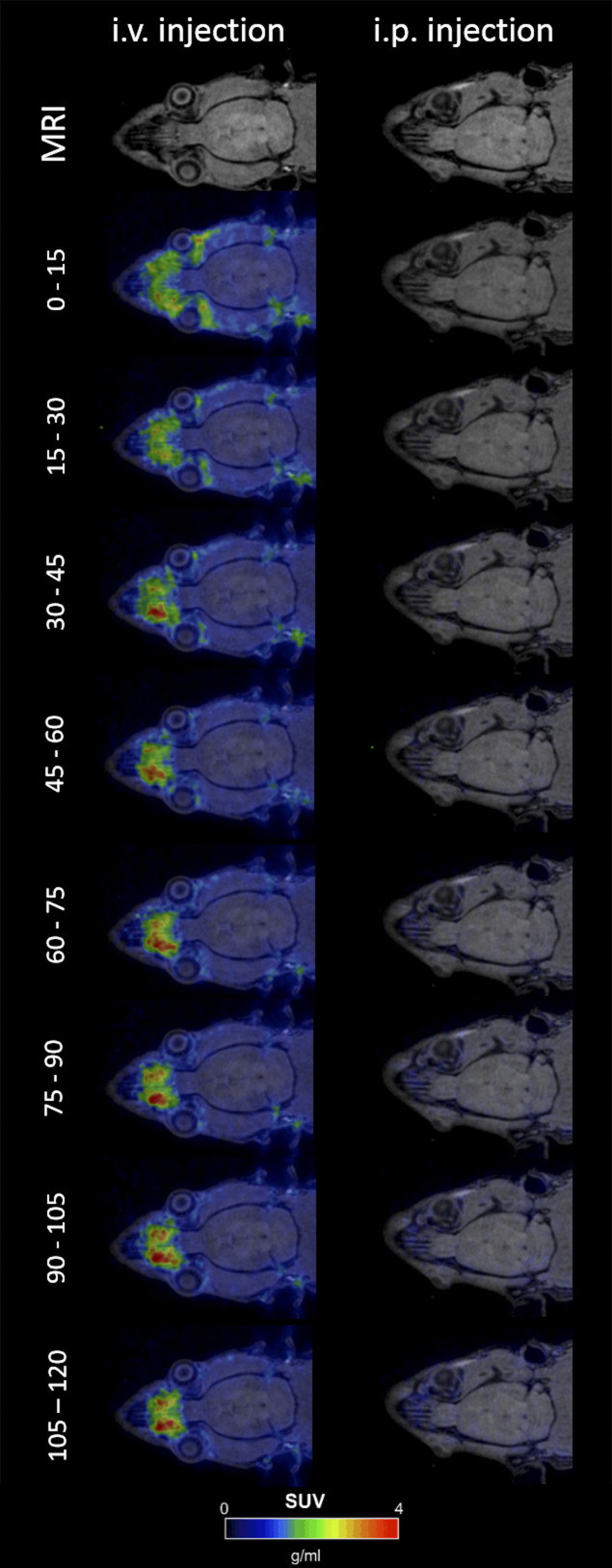
Intravenous injection causes fast ^64^Cu signals in brain proximate vessels. Positron emission tomography scans of wild-type rats i.v. injected with ^64^Cu show its distribution in large blood vessels in the brain proximity already 15 min post injection and a subsequent time-dependent decrease of the positron emission tomography signal. In contrast, i.p. injection of copper causes very low signal intensities over 120 min.

Currently, chelation is the main therapeutic approach in WD to avoid copper toxicity upon its accumulation occurring primarily in the liver. However, if chelators mobilize excess hepatic copper, this may cause increased blood copper. Although such mobilization is a prerequisite for renal clearance of chelated copper, it could nevertheless lead to potentially undesirable systemic copper effects, for example, “neurological worsening” as suggested by DPA treatment studies in toxic milk mice (9). Indeed, upon intravenous ^64^Cu-injection, within minutes the metal can be largely traced by positron emission tomography (PET) in brain supporting vessels in wild-type rats (Fig 1A). In contrast, by intraperitoneal ^64^Cu-injection, that is, mimicking nutritional uptake, copper is not detected in brain by PET, even hours later (Figs 1A and S1).

We therefore investigated if or to what extent copper appears in serum in untreated *Atp7b*^*+/−*^ control and *Atp7b*^*−/−*^ rats (alternatively termed WD rats), but also in *Atp7b*^*−/−*^ rats treated with either DPA or ALXN1840 (Fig 1B). As in WD patients, WD rats lack copper incorporation into ceruloplasmin (39), and therefore untreated *Atp7b*^*−/−*^ animals have a very low serum copper level. Treatment of WD rats with ALXN1840 resulted in a significant increase in serum copper levels (likely due to ALXN1840–Cu–albumin tripartite complex formation, see below). This did not occur with DPA treatment (Fig 1B). As this latter absence may be due to fast renal copper clearance, we next investigated the excretion of copper via urine (Fig 1C), but also feces (Fig 1D). Whereas untreated *Atp7b*^*+/−*^ and *Atp7b*^*−/−*^ rats had low copper levels in either urine or feces collected over 24 h, DPA treatment of WD rats led to a significantly increased copper excretion into urine, in agreement with typical diagnostic findings in WD patients. In contrast, no profoundly elevated net copper excretion was noted upon ALXN1840 treatment under the chosen conditions (i.e., an observation period of 96 h (40)). Thus, these chelators elevate blood copper levels to different extent. Whereas in the case of DPA a rapid renal clearance (blood peak between 1 and 3 h after application (41)) may have avoided the detection of elevated serum copper levels here, it was significantly elevated upon ALXN1840 treatment in WD rats.

### ALXN1840 forms a stable complex with copper and albumin

**Figure 2.**
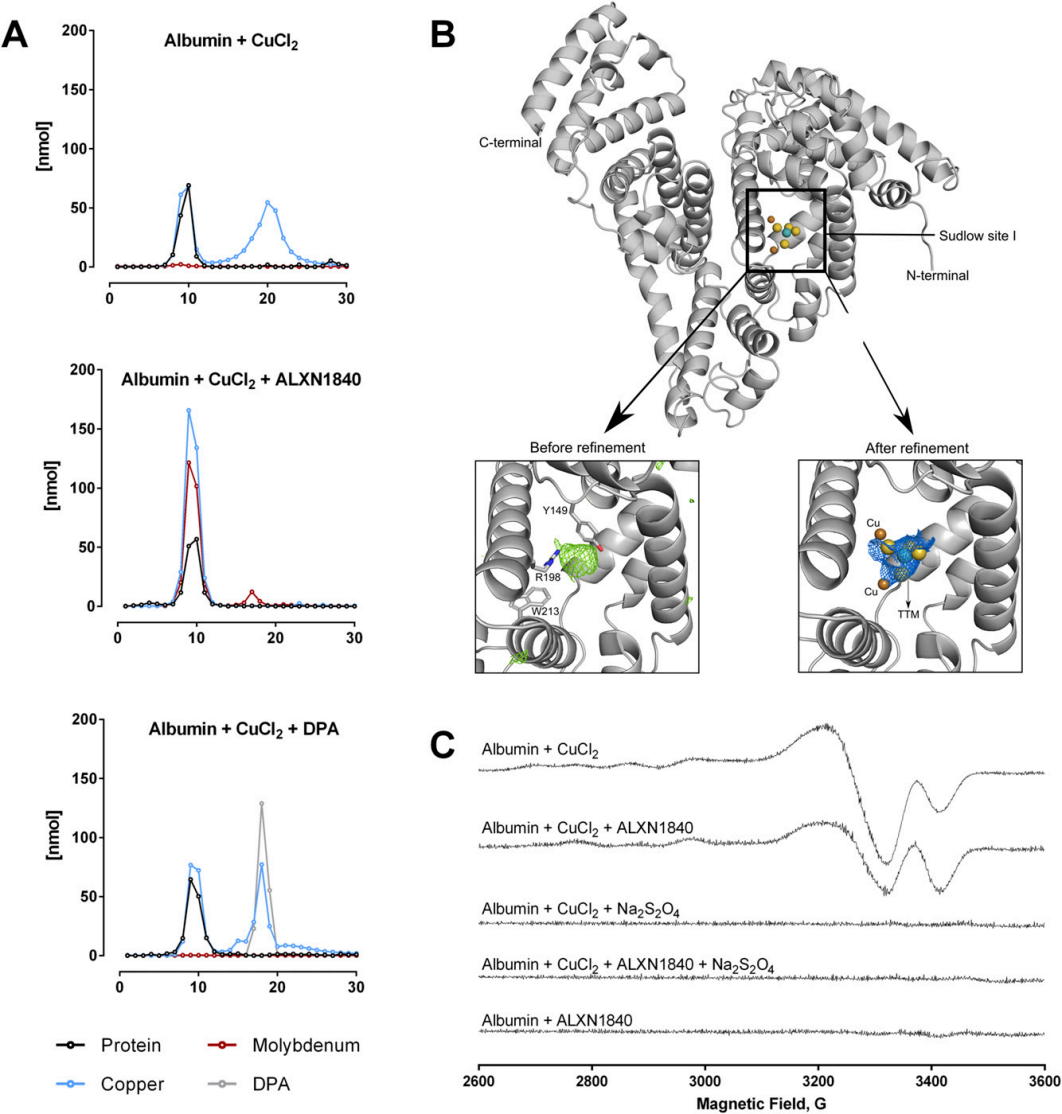
ALXN1840 forms a stable complex with albumin and copper. **(A)** Size-exclusion chromatography demonstrates that a Cu–albumin mixture of a molar ratio of 3:1 causes the formation of a Cu–albumin complex as well as a second peak representing unbound copper. In the additional presence of ALXN1840, a single peak is encountered, suggesting the formation of an albumin–Cu–ALXN1840 complex, in contrast to the addition of DPA (N = 2). **(B)** Structural analysis of albumin (upper panel) and its Sudlow site I (SsI). The lower panels present close-ups of SsI with calculated difference map (F_obs_–F_calc_, colored green) before (left) and after (right) refinement. ALXN1840 and copper atoms are covered by calculated 2F_obs_–F_calc_ map (colored blue), indicating the presence of these molecules inside SsI. **(C)** Electron paramagnetic resonance measurements reveal a partial reduction of Cu^2+^ in the albumin/Cu/ALXN1840 tripartite complex. Complete Cu^2+^ reduction is achieved by excess sodium dithionite (Na_2_S_2_O_4_).

In WD, blood copper may be loosely bound to albumin (14). In fact, upon mixing 750 μM copper with 250 μM albumin (i.e., a molar ratio 3:1), a subsequent gel filtration removed about half to two thirds of the copper from albumin (Fig 2A top panel). Clearly, such easily removable loosely bound copper may present a potential threat when present in WD patients’ blood. Moreover, when incubated with DPA (at a molar ratio Cu–albumin-DPA of 3:1:3), a portion of the copper pool stayed with albumin, likely due to DPA’s lower copper affinity in comparison to the high-affinity albumin binding site (K_d_ (DPA) = 2.4 × 10^−16^ M (38) versus K_d_ (N terminus of albumin) = 6.7 × 10^−17^ M (19)), whereas the residual copper co-migrated with DPA (Fig 2A lower panel). Therefore, it appears that the capacity of DPA to de-copper albumin is limited to the loosely bound copper at the applied molar ratios.

Intriguingly, when co-incubated with ALXN1840 (K_d_ = 2.3 × 10^−20^ M (38)), which has an affinity for copper that is magnitudes greater than that of DPA or albumin, albumin was not fully de-coppered but rather one prominent gel filtration peak appeared (Fig 2A middle panel), comprising the protein and large parts of copper as well as ALXN1840 (detected as molybdenum). This feature has been described for ALXN1840 in man (35) or for TTM in LEC rats (TTM is the active molecule in ALXN1840) (42, 43, 44, 45) and is due to the formation of a Cu–albumin–ALXN1840/TTM complex, previously termed the “tripartite complex” (TPC).

How is copper bound to the TPC? The single TPC gel filtration peak clearly indicated its tight binding to the protein. Consequently, we used X-ray crystallography of the TPC (Fig 2B) and, upon data refinement, we could track ALXN1840 with two bound copper ions in the so-called Sudlow Site 1, a profound cleft in albumin formed by His241, Tyr149, Arg256, Lys237, and Ala290 (Fig 2B). Thus, in the presence of albumin, copper and ALXN1840 get deeply embedded into the protein. This finding may explain the lack of urinal copper excretion in ALXN1840-treated WD rats (Fig 1C), as renal albumin clearance is very limited (46). In addition, electron paramagnetic resonance (EPR) studies demonstrated a change in copper redox status in the TPC versus Cu–albumin (Fig 2C). Whereas the latter revealed the typical cupric Cu(II) signal (47), in the presence of ALXN1840, the signal intensity of the EPR-active cupric copper dropped by around 50%, suggesting the reduction of one cupric copper ion to EPR-silent cuprous Cu(I) (Fig 2C second trace). Indeed, upon addition of sodium dithionite that fully reduces Cu(II) to Cu(I), no EPR signal was detected in both, Cu–albumin and TPC (Fig 2C lower traces).

In summary, using the above molar ratios, only the tightly bound copper stays with albumin, whereas more loosely bound copper is either set free upon gel filtration, bound to DPA, or relocated to the Sudlow site of albumin by ALXN1840 forming the TPC. Thus, if excess liver copper appears in blood in untreated WD, it represents a potential toxic threat to secondarily affected tissues like the brain, but this situation may change depending on the particularly used chelator.

### High-affinity chelation prevents Cu–albumin–induced cell toxicity

**Figure 3.**
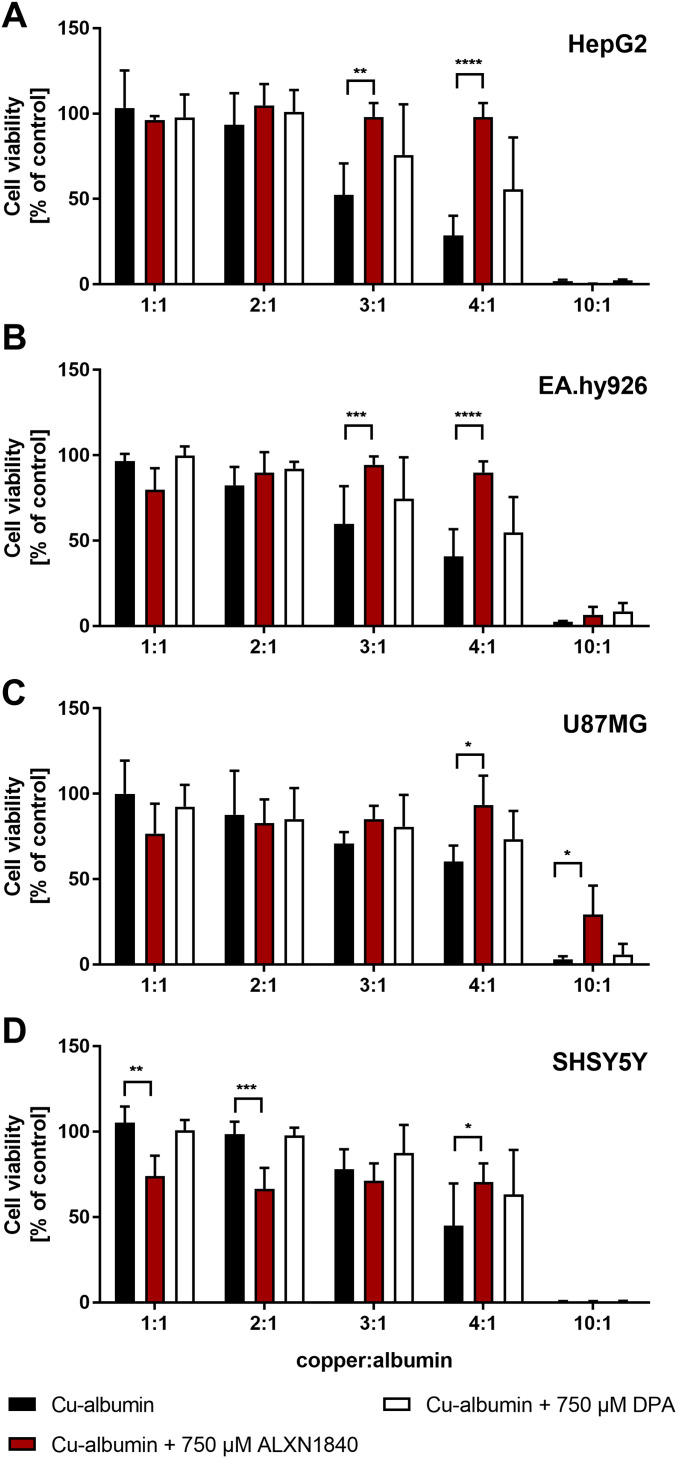
Cu–albumin ratio dependent toxicity. **(A, B, C, D)** Increasing molar Cu–albumin ratios cause a ratio dependent decrease in CellTiter-Glo-assessed cell viability in (A) HepG2, (B) EA.hy926, (C) U87MG, and (D) SHSY5Y cells. Such cytotoxicity is largely avoided by ALXN1840 but to a very minor part by DPA (both 750 μM, N = 3–5, n = 6–10). Two-way ANOVA with Dunnett’s multiple comparisons test was used for statistical analysis. *P < 0.05, **P < 0.01, ***P < 0.001, ****P < 0.0001.

**Figure S2.**
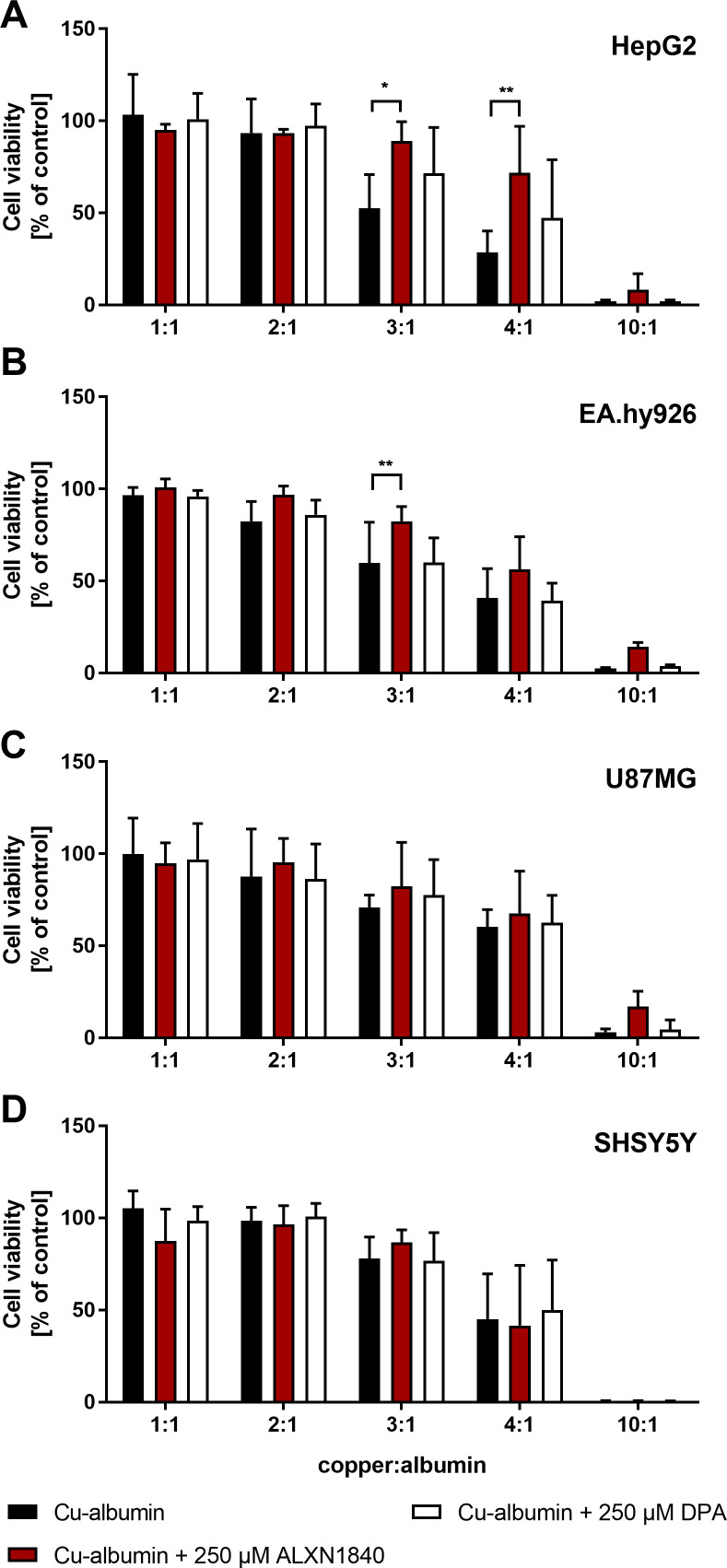
Partial rescue of Cu–albumin induced cell toxicity. **(A, B, C, D)** A low dose of ALXN1840 (250 μM) partially rescues Cu–albumin–induced cell toxicity in (A) HepG2 and (B) EA.hy926 cells, but not (C) U87MG and (D) SHSY5Y cells (CellTiter-Glo assay) in contrast to low-dose DPA (N = 3–5, n = 6–10). Two-way ANOVA with Dunnett’s multiple comparisons test was used for statistical analysis. *P < 0.05, **P < 0.01, ***P < 0.001, ****P < 0.0001.

To demonstrate loosely albumin-bound copper toxicity, we tested hepatic HepG2 cells (human hepatocellular carcinoma) (Figs 3A and S2A) and surrogate brain cell types, such as EA.hy926 (human endothelium) (Figs 3B and S2B), U87MG (human astrocytoma) (Figs 3C and S2C) and SHSY5Y (human neuroblastoma) (Figs 3D and S2D) cells for their vulnerability against Cu–albumin. Albumin was at a concentration of 250 μM and increasing molar ratios of Cu versus albumin (1:1–10:1) were used to mimic its progressive copper load. Whereas at a molar ratio of 1:1, copper is rather tightly bound to albumin, at higher ratios (especially when ≥3:1) an increasing amount of loosely bound copper is available (exemplarily shown in Fig 2A top panel for a ratio of 3:1).

**Figure S3.**
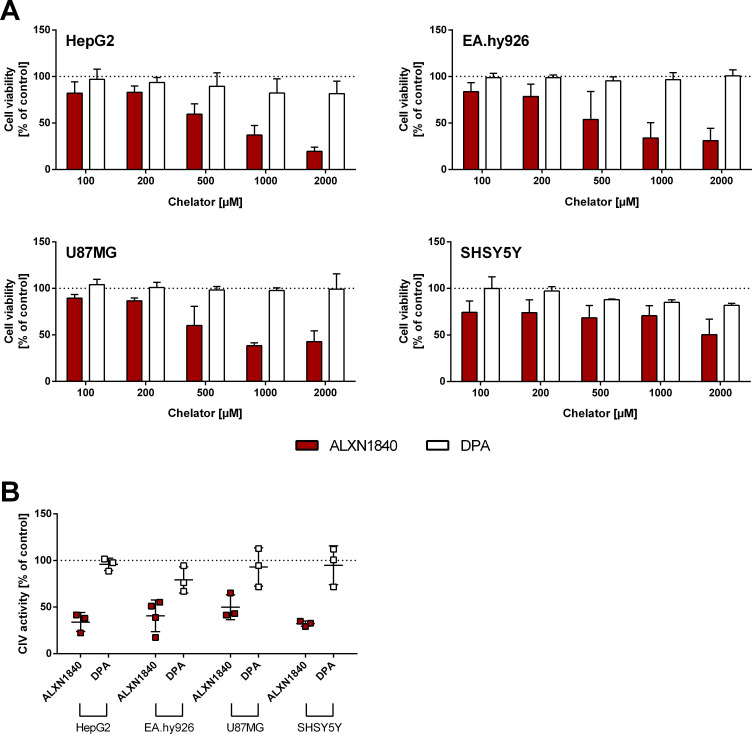
High-affinity chelators may be cell- and mitochondria-toxic in the absence of copper accumulations. **(A)** In the absence of Cu–albumin, all investigated cell lines show a dose-dependent reduction in cell viability (CellTiter-Glo) by the high-affinity chelator ALXN1840, but not by DPA (N = 3, n = 6). **(B)** Complex IV activity is already reduced at a non-toxic ALXN1840 concentration (200 μM) in all cell lines, whereas 200 μM DPA has no effect on complex IV activity (N = 3–4, n = 6–10).

After 24 h of incubation, all tested cell lines demonstrated cellular toxicity against a dose-dependent increase in loosely albumin-bound Cu, as assessed by the CellTiter-Glo assay (Fig 3A–D, black bars). Importantly, the high-affinity chelator ALXN1840 at a concentration of 750 μM fully avoided toxicity up to a Cu–albumin ratio of 4:1 (Fig 3, red bars). In contrast, DPA was much less effective. Only in HepG2 cells, and only at a high 750 μM dose, a modest rescuing effect was observed upon DPA addition (Figs 3A and S2A, white bars) that was, however, completely absent, in, for example, endothelial EA.hy926 cells. Despite this admittedly artificial (with respect to total copper amount and observed time frame of 24 h for toxicity) testing scenario, these results nevertheless suggest that endothelial cells may be particularly vulnerable to Cu–albumin and that DPA cannot block this Cu toxicity. Importantly, the lack of DPA rescue was not due to toxicity of DPA itself, as in a copper-free setting even DPA concentrations up to 2 mM were found to be non-toxic (Fig S3A). However, as a note of caution, at such settings, i.e. without external Cu–albumin addition, rather the high-affinity chelator ALXN1840 becomes toxic at elevated concentrations (Fig S3A), possibly because of an interference with copper containing vital enzymes such as the cytochrome *c* oxidase (Fig S3B).

### ALXN1840 may prevent copper toxicity because of its high copper affinity

**Figure 4.**
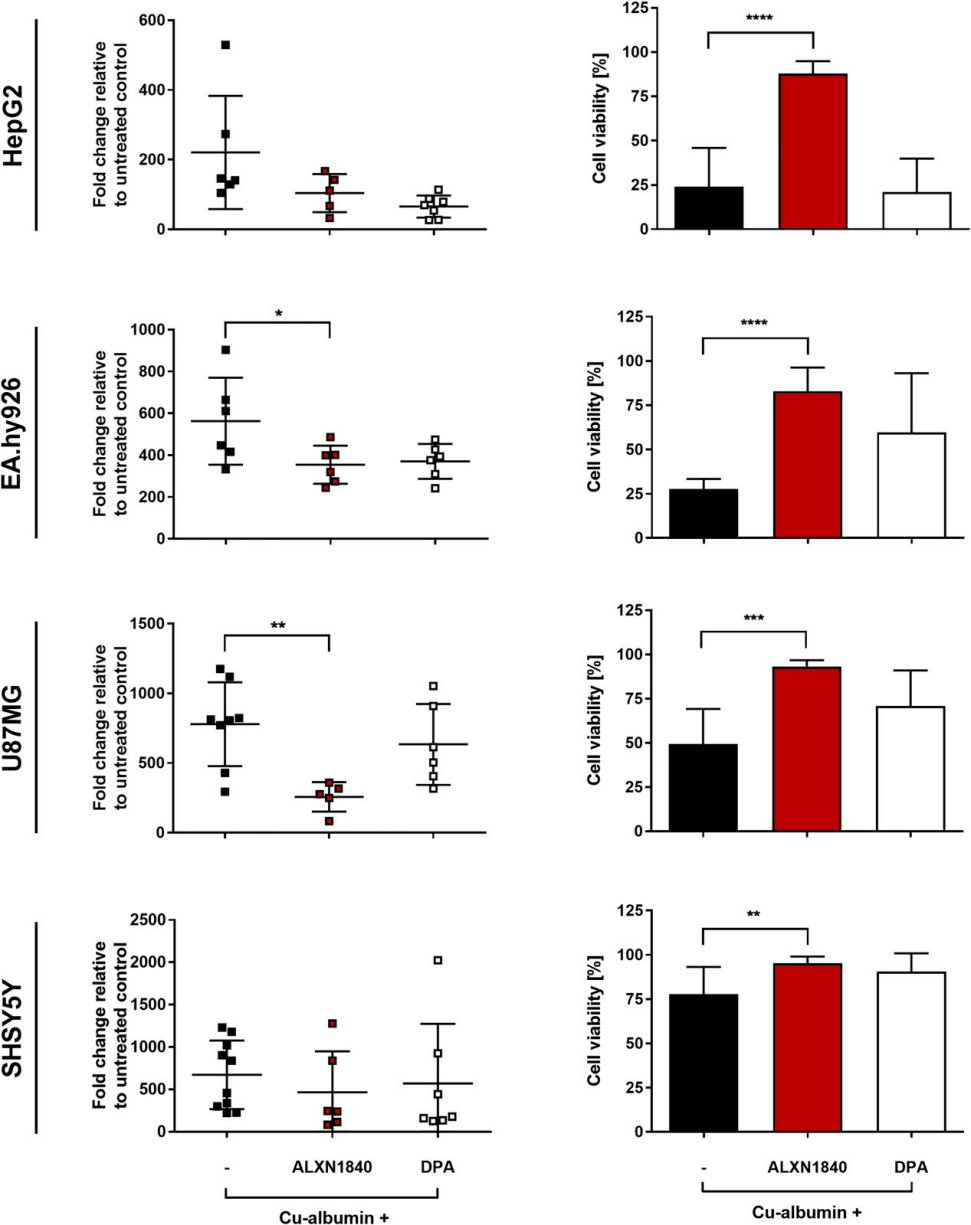
Massive cellular copper accumulations are partially resolved by ALXN1840. (Left panels) Cu–albumin incubation at a molar ratio of 3:1 (i.e., 750 μM Cu^2+^ and 250 μM albumin) leads to massive copper accumulation in all investigated cell lines. In the co-presence of ALXN1840, U87MG, and EA.hy926 cells, but not HepG2 and SHSY5Y cells, present with significantly lower copper content, not observed in the co-presence of DPA (N = 4–12). One-way ANOVA with Dunnett’s multiple comparisons test was used for statistical analysis. (Right panels) Such Cu–albumin incubations lead to massive cell viability loss of HepG2, EA.hy926, U87MG, and SHSY5Y as assessed by trypan blue staining. Co-presence of ALXN1840, but not of DPA, significantly protects all tested cell lines (N = 4–12). One-way ANOVA with Sidak’s multiple comparisons test was used for statistical analysis. *P < 0.05, **P < 0.01, ***P < 0.001, ****P < 0.0001.

As can be seen in Fig 4, a profound dose of Cu–albumin (here at a ratio of 3:1) increased the cellular copper content more than 100-fold in all tested cell types (Fig 4, left panels), paralleled by massive cell death, with SHSY5Y cells being the least and EA.hy926 and HepG2 cells being the most affected (Fig 4 right panels). This copper toxicity could not be avoided even by high doses of DPA, that is, equimolar to copper (Figs 3 and 4, right panels). Moreover, a significant de-coppering was not noted in any of the cell lines upon DPA co-treatment despite a tendency for lower copper content in EA.hy926 and SHSY5Y cells (Fig 4, left panels). In remarkable contrast, co-treatment with the high-affinity chelator ALXN1840 significantly decreased the copper content in EA.hy926 and U87MG cells, and cellular viability was significantly maintained (Fig 4, right panels). These data indicate that the cell viability protection exerted by the high-affinity chelator, however, is at best only in part due to its capacity to lower the cellular copper content. In HepG2 cells, for example, highly similar cellular copper contents were found in cells either co-treated by ALXN1840, DPA, or treated by Cu–albumin alone (Fig 4, upper left panel). Despite this equal copper burden, ALXN1840 rescued HepG2 cells, whereas DPA did not (Fig 4, upper right panel). It therefore appears much more plausible that it is the enormous copper affinity of ALXN1840 (K_d_ ≈ 10^−20^) that avoided copper toxicity by its tight binding whether out- or inside cells. Indeed, even the highest known copper affinities of potential cellular binding partners are orders of magnitude lower (K_d_ ≈ 10^−16^ (48)). In contrast, as its dissociation constant is exactly in this latter range, this may also explain why DPA was unable to ensure or only tendentiously increased cellular viability.

### ALXN1840 ameliorates Cu–albumin–induced mitochondrial damage

**Figure S4.**
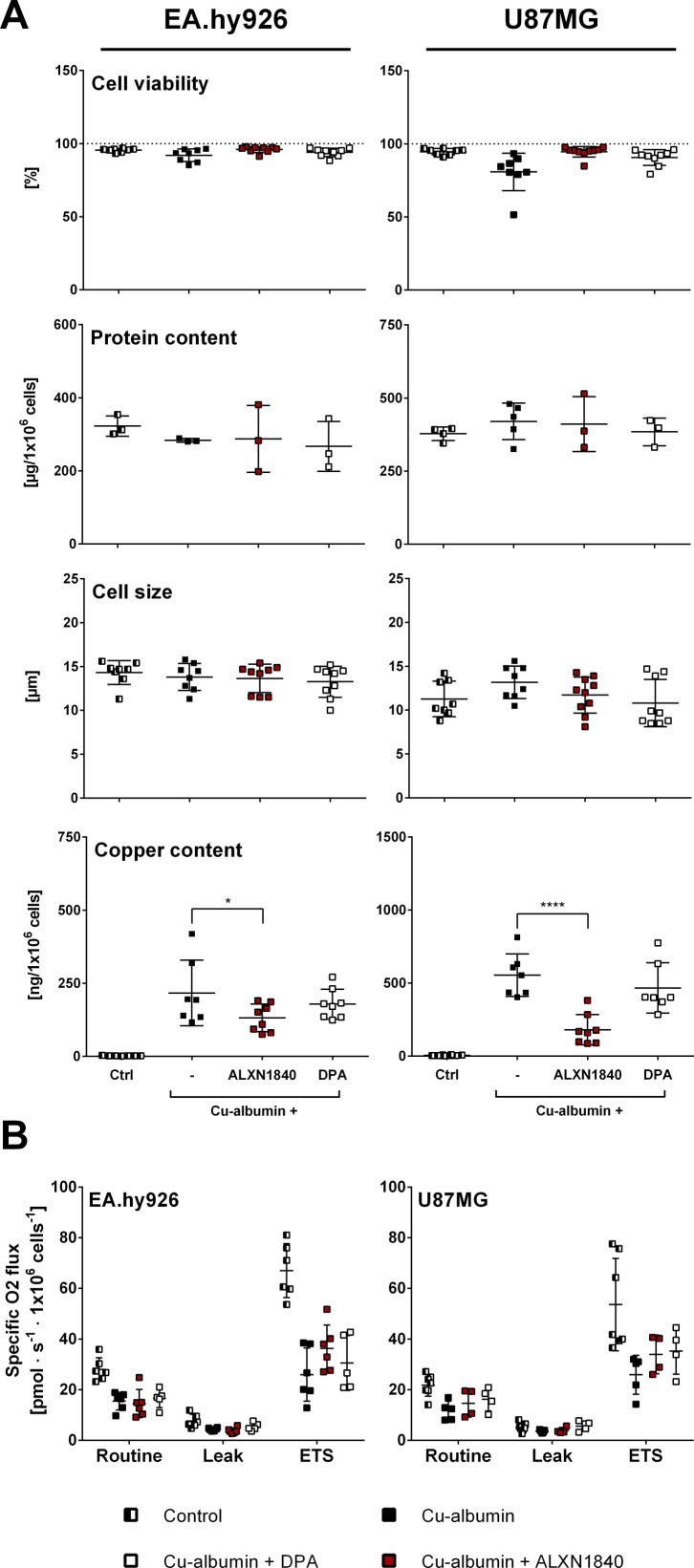
Cellular parameters of EA.hy926 and U87MG cells subjected to high-resolution respirometry measurements. **(A)** Cell viability assessed by trypan blue (N = 5–13), cell size (N = 5–13), and cellular protein (N = 3, n = 9) content of EA.hy926 and U87MG cells are comparable in the investigated cells. In contrast, cellular copper content is strongly elevated upon Cu–albumin treatment, partially depleted by co-presence of ALXN1840 (N = 5–13). **(B)** Mitochondrial respiration is decreased in EA.hy926 and U87MG cells upon Cu–albumin incubation, partially rescued by co-presence of (N = 4–7; electron transport system, capacity of the electron transport system). Two-way ANOVA with Dunnett’s multiple comparisons test was used for statistical analysis. *P < 0.05, **P < 0.01, ***P < 0.001, ****P < 0.0001.

In WD, reports have amply demonstrated that copper severely affects hepatocyte mitochondria (49, 50, 51). Only very recently have comparative studies shown a high copper sensitivity of brain mitochondria (52). Here, we have specifically looked to evaluate whether Cu–albumin could also impose structural and/or functional damage on mitochondria in cells that constitute the BBB, that is, endothelial cells and astrocytes. This was foremost because a clinical report had suggested copper-induced BBB damage to occur in neurologic WD patients (27). To exclude mitochondrial impairment as secondary effect of copper-induced cell demise, we adjusted the Cu–albumin concentration (ratio 3:1) such that cell viability was comparable with untreated controls (Fig S4A). Besides, neither cellular protein content nor cell size were affected by such settings that, however, caused an enormous increase in cellular copper content with respect to untreated controls (Fig S4A).

**Figure 5.**
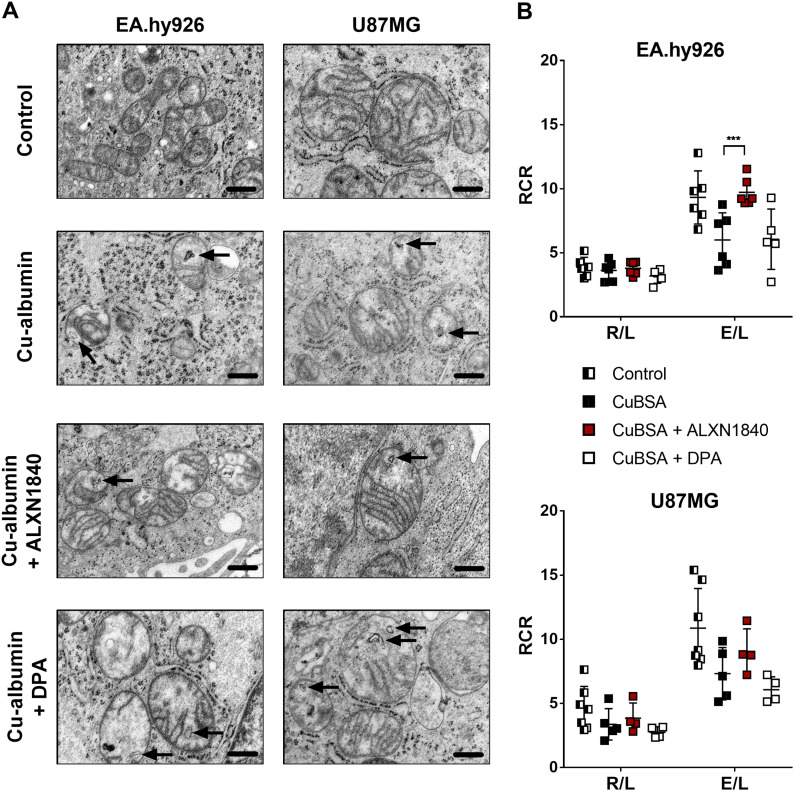
Cu–albumin–induced structural and functional mitochondrial alterations. **(A)** Cu–albumin incubation causes membranous inclusions and unorganized/shortened cristae in mitochondria of EA.hy926 and U87MG cells. In the co-presence of ALXN1840, but not of DPA, these alterations are partially resolved (Scale bars 500 nm). **(B)** Respiratory control ratios (RCR), defined as routine to leak respiration (R/L) or electron transport system to leak respiration (E/L). Co-presence of ALXN1840, but not of DPA, significantly/markedly augments the Cu–albumin induced E/L ratio drop in EA.hy926 and U87MG cells, respectively (N = 4–7). Two-way ANOVA with Dunnett’s multiple comparisons test was used for statistical analysis. *P < 0.05, **P < 0.01, ***P < 0.001, ****P < 0.0001.

Electron micrographs of Cu–albumin versus untreated cells demonstrated prominent mitochondrial structural alterations in EA.hy926 cells, and present, but more modest, alterations in U87MG cells (Fig 5A). A loss or structural disorientation of the mitochondrial cristae and membranous inclusions were observed (arrows in Fig 5A). Importantly, ALXN1840 co-treatment partially avoided these structural abnormalities, demonstrating mitochondria with electron-dense matrices and structured cristae similar to untreated control cells. In contrast, DPA was of no/minor effect as mitochondria presented with short and unstructured cristae and membranous inclusions (Fig 5A).

These structural deficits were paralleled by functional mitochondrial impairments (Fig 5B), which were especially apparent in high-resolution respiratory measurements of treated cells under fully uncoupled conditions (i.e., forcing mitochondria to maximal respiration, electron transport system [ETS], Fig S4B). When calculating the respiratory control ratios (the paradigm markers for mitochondrial integrity and functionality) by dividing either the “routine” (R, i.e., in presence of ADP) or the fully uncoupled state (ETS, i.e., upon titration with carbonyl cyanide m-chlorophenyl hydrazine (CCCP)) oxygen consumption rate by the so-called leak state (L, i.e., respiration w/o ADP), especially the E/L ratio demonstrated clear mitochondrial bioenergetic deficits, that again could, either significantly in the case of EA.hy926 cells or tendentiously in the case of U87MG cells, be avoided by the presence of ALXN1840, but not by DPA (Fig 5B).

### Disruption of the tight endothelial cell layer of the BBB by Cu–albumin is prevented by ALXN1840, but not by DPA

Human EA.hy926 endothelial (and U87MG astrocytoma) cells were highly vulnerable to increasing Cu–albumin challenges (Fig 3B) and demonstrated prominent mitochondrial structural and functional deficits (Fig 5). We thus reasoned that especially the endothelial cell layer that requires mitochondrial integrity and functionality for remaining tightly sealed within the BBB (53, 54), may constitute a pivotal target of Cu–albumin toxicity.

Consequently, we used a well-characterized *in vitro* model of the endothelial BBB using primary porcine brain capillary endothelial cells (PBCECs) cultivated on Transwell inserts (55, 56). As in the BBB, these primary cells form a mono-cellular tight epithelial barrier, as can be biophysically assessed by the continuous measurement of their transepithelial electrical resistance (TEER) and their monolayer capacitance as measure for cellular integrity (57).

**Figure S5.**
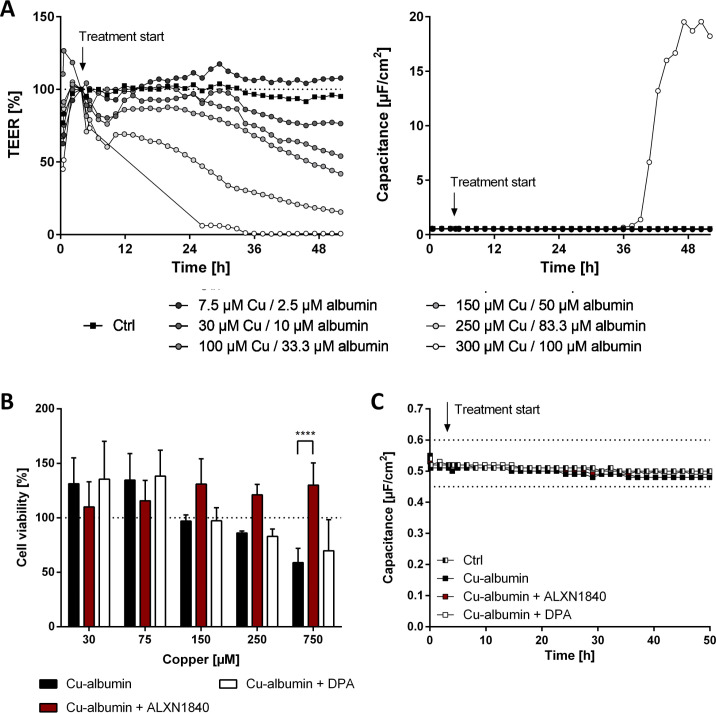
Cu–albumin causes a dose-dependent leakiness of PBCEC monolayers already at non–cell-toxic copper concentrations. **(A)** Exemplary curves of transendothelial electrical resistance (TEER) and capacitance changes of PBCEC monolayers in the presence of increasing Cu–albumin (Cu/albumin ratio 3:1 in all cases). Already low Cu–albumin concentrations cause progressive TEER decreases, whereas a capacitance increase, indicative of cell death, is only detectable at the highest tested Cu–albumin concentration. **(B)** Neutral red assay of PBCECs reveals no toxicity below 250 μM copper and 83.3 μM albumin upon 48 h of incubation. However, 750 μM copper (and 250 μM albumin) causes reduction in cell viability, which can be rescued by the presence of 750 μM ALXN1840 (N = 3, n = 12). **(C)** Capacitance values of PBCEC monolayers are unaffected by Cu–albumin treatment in the absence or presence of ALXN1840 or DPA (N = 2, n = 4).

First, we validated that increasing Cu–albumin concentrations (all at a molar ratio of 3:1) progressively decreased the TEER (Fig S5A). Of note, only the highest used Cu–albumin concentration (300 μM copper/100 μM albumin) caused a massive capacitance increase, that is, cell death, after 36 h of incubation (Fig S5A) that was additionally validated via the neutral red assay (Fig S5B). Thus, the leakiness of the endothelial cell layer of the BBB induced by lower Cu–albumin concentrations is not due to the mere induction of cell death but is a clear sign of endothelial cell stress.

**Figure 6.**
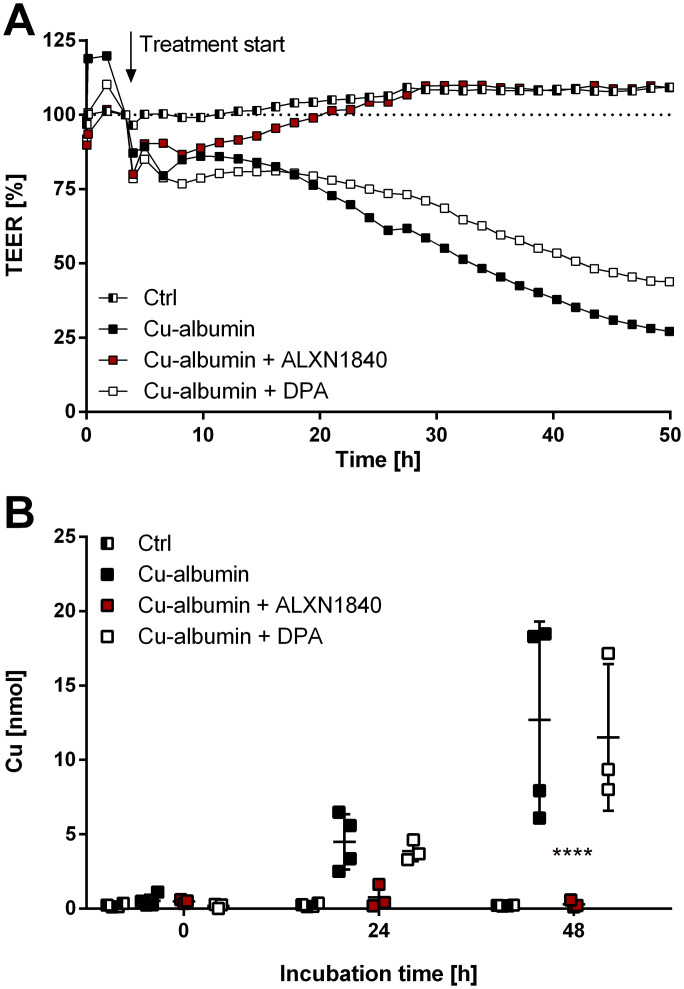
Cu–albumin permeabilizes blood–brain barrier constituting endothelial cell monolayers. **(A)** Cu–albumin (250 μM copper, 83.3 μM albumin), either alone or in the co-presence of DPA, leads to a time-dependent reduction in the transepithelial electrical resistance (TEER) of primary porcine brain capillary endothelial cell monolayers that is avoided by the co-presence of 250 μM ALXN1840 (N = 2, n = 4). **(B)** Such decreased resistance is paralleled by progressive copper appearance in the basolateral compartment (resembling the brain parenchyma) (N = 2, n = 4). Two-way ANOVA with Dunnett’s multiple comparisons test was used for statistical analysis. *P < 0.05, **P < 0.01, ***P < 0.001, ****P < 0.0001.

We subsequently determined the capability of the copper chelators ALXN1840 and DPA to prevent such Cu–albumin–induced endothelial BBB damage (Fig 6). Thereto, a Cu–albumin concentration (250 μM Cu/83.3 μM albumin, molar ratio of 3:1) was chosen that readily caused the BBB to become leaky (i.e., decrease the TEER, Fig 6A), but did not induce cell death within the observed time frame as evidenced by time-stable capacitance of the PBCEC monolayers (Fig S5C). Importantly, DPA co-treatment was not able to prevent the copper-induced TEER loss (Fig 6A). In contrast, PBCEC monolayers treated with Cu–albumin in the presence of ALXN1840 demonstrated stable TEER values for 48 h, indistinguishable from untreated monolayers (Fig 6A). This was further validated by determining the copper influx into the basolateral compartment of the Transwell system that is not directly accessible in tight PBCEC monolayers (control in Fig 6B). In fact, copper influx progressively occurred upon Cu–albumin treatment that could not be avoided by DPA but was fully avoided by ALXN1840 co-treatments (Fig 6B). Thus, elevated Cu–albumin causes leakiness of the endothelial BBB layer already in the absence of endothelial cell death that is associated with copper influx into the otherwise shielded compartment, and this can be avoided by the presence of ALXN1840, but not by DPA.

**Figure 7.**
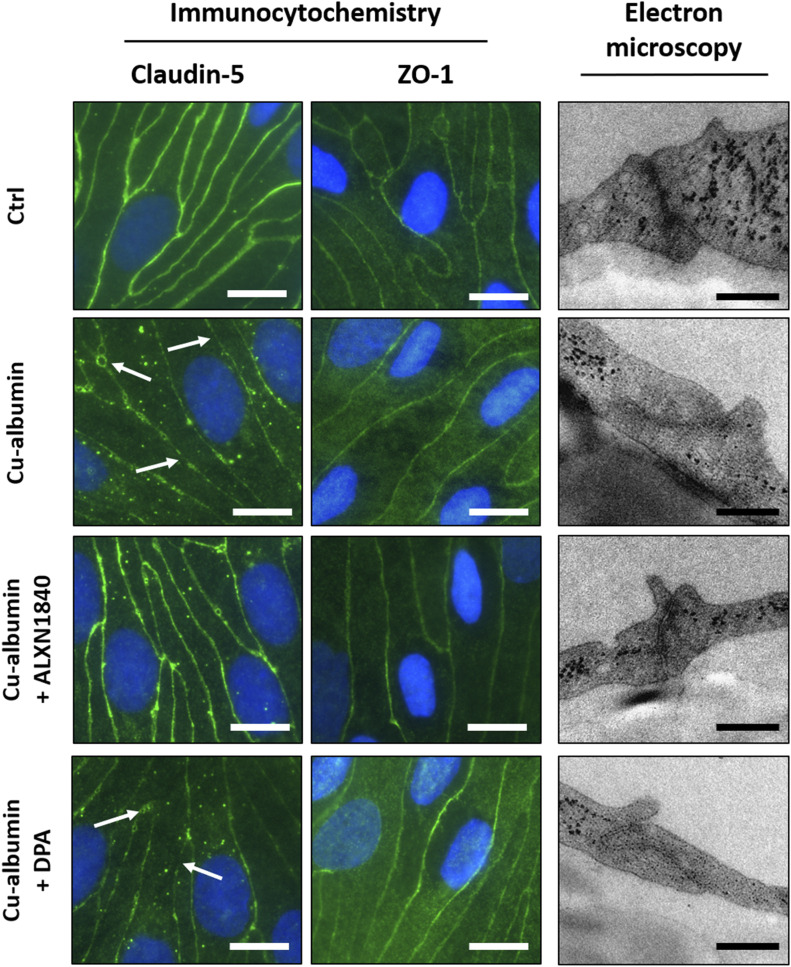
Cu–albumin disrupts tight junctions in blood brain barrier constituting endothelial cell monolayers. (Left panels) Immunocytochemistry staining against the tight junction protein Claudin-5 shows a continuous staining of the cell margins in control PBCECs, being disrupted upon Cu–albumin treatment (250 μM copper and 83.3 μM albumin). Co-presence of ALXN1840 (250 μM), but not of DPA, alleviates these morphologic alterations. (Middle panels) The tight junction–associated protein Zonula occludens-1 (ZO-1) reveals a plasma membrane associated or more diffuse cytosolic localization in either untreated control or Cu–albumin–treated PBCECs, respectively. Co-presence of ALXN1840, but not of DPA, avoids such diffuse localization. Scale bars equal 10 μm. Electron micrographs of Cu–albumin–treated versus control PBCECs reveal less electron-dense tight junction structures. Tight junctions appear electron dense upon co-presence of ALXN1840 but not of DPA. Scale bars equal 250 nm.

Finally, Cu–albumin–induced PBCEC monolayer damage can be visualized by either immunocytochemistry or electron microscopy (Fig 7). First, Claudin-5, an integral membrane protein of tight junction strands (58), demonstrated a continuous and uninterrupted distribution at the cell margins in untreated control cells. In contrast, Cu–albumin–treated PBCECs displayed gap formations between cells as well as serrated and diffuse Claudin-5 presence. Of note, this structural damage happens already at incubation conditions that do not elicit cell toxicity/death, that is, that do not kill the cells as their intact nuclei can be seen in the immunofluorescence (Fig 7) and also biophysically confirmed by a lack of capacitance increase (Fig S5A). In the presence of ALXN1840, Claudin-5 expression was continuous and uninterrupted, whereas the presence of DPA could not prevent copper-induced gap formation (Fig 7, left panels). Second, Zonula occludens-1 (ZO-1), an intracellular tight junction-associated protein (59), appeared continuously present and uninterrupted at the cell borders in untreated control PBCEC monolayers (Fig 7, middle panels). Upon Cu–albumin treatment, a pronouncedly more diffuse staining of ZO-1 occurred that could be fully protected by ALXN1840 co-treatment, but not by DPA (Fig 7, middle panels). Such a protein loss at the tight junctions was also apparent from, third, electron micrographs. In control PBCEC monolayers, because of deposition of the contrasting agent at protein-rich moieties, these structures appear electron dense. Upon Cu–albumin treatment, however, these structures were much more electron permissive, not protected for by DPA, but by ALXN1840 (Fig 7, right panels).

## Discussion

In this study, we have demonstrated that intravenously present copper can easily access the brain supporting vessels (Fig 1) and that upon increase, copper is progressively loosely bound to serum albumin (Fig 2). Such Cu–albumin is cell-toxic (Figs 3 and 4) and endothelial cells that constitute the tight barrier to protect the brain are especially vulnerable. At Cu–albumin concentrations that do not exert immediate cell death, mitochondria are a vulnerable target (Fig 5) and affected cells of the endothelial barrier demonstrate leaky tight junctions, resulting in a progressive copper cross-transition (Figs 6 and 7). Importantly, all these features were largely avoided by co-treatment with the high-affinity copper chelator ALXN1840, but not with DPA (Figs 2, 3, 4, 5, 6, and 7).

How do these findings relate to *in vivo* situations in WD patients, where 18–68% have been reported to suffer from neurological symptoms, for example, tremor, dysarthria, and dystonia (13), and their disease severity has been suggested to correlate with increased available serum copper?

First, massively elevated copper brain levels have been observed in WD patients. This may be realized as counterintuitive because serum albumin has an enormous capacity to tightly bind the metal at a binding site with very high affinity. Moreover, blood copper concentrations in neurologic WD patients are considerably lower than albumin’s binding capacity. A possible explanation for how copper could nevertheless slowly accumulate in the brain may be a constant competition for Cu between serum albumin and the copper uptake transporter CTR1 at the BBB, which would result (after years/decades) in brain damage. The other not mutually exclusive possibility is repeated focal hepatocyte death in the liver to cause repetitive intense copper pulses in the blood that may overwhelm the serum albumin binding capacity, thus allowing copper uptake into brain. Indeed, in *Atp7b*^*−/−*^ rat livers, copper is not evenly distributed but rather present in “copper hotspots” with 3.5 times higher copper concentration than the surrounding liver tissue (60). Demise of such hotspots could possibly result in transient copper pulses. In a collaborative effort, we have very recently determined an extractable serum Cu of 4.0 ± 2.3 μM in healthy control rats, 2.1 ± 0.6 μM Cu in healthy *Atp7b*^*−/−*^ rats, and 27 ± 16 μM Cu in diseased *Atp7b*^*−/−*^ rats, using a Cu-specific column that separates bound Cu from extractable Cu (61). In agreement with these data, we did not see elevated urinary copper excretion in untreated but still healthy *Atp7b*^*−/−*^ rats similar to controls (Fig 1C), but in contrast to findings in WD patients. Thus, it is only upon hepatocyte death in the *Atp7b*^*−/−*^ rats (i.e., when they become diseased) that there are up to 10-fold higher amounts of “free” copper in serum that may be taken up by the brain. However, when hepatitis starts animals die shortly after and before developing neuronal symptoms, thereby precluding the study of neurological deficits in these animals. Furthermore, as ATP7B is present in the blood-facing membrane of the cerebral endothelium (24), mutations in ATP7B could lead to a reduced/blocked re-transport of excessive copper into the blood, thereby causing a one-way entry of the metal into the brain parenchyma. Here, only ATP7A-mediated copper transport via the cerebrospinal fluid back into the systemic circulation would allow lowering brain copper (24). Taken together, these results would explain the observed correlation in WD patients of neurological severity and increasing NCC correlating with such insults (7). Perhaps, the strongest indication for such a “copper pulse” scenario is the worsening of neurological symptoms observed in up to 50% of neurologic WD patients seen shortly after starting the treatment with the copper chelator DPA (28, 29, 30, 31, 32, 33, 34). This unwanted drug effect was attributed to the (abrupt) mobilization of copper from the liver into the bloodstream, thereby leading to an increased copper accumulation in the brain (9). Indeed, in the WD animal model toxic milk mouse, there is some experimental evidence for this viewpoint. Upon intragastric D-penicillamine (DPA) administration, within days, a significant increase in copper in serum and also in brain was demonstrated in these mice (9), and thus one may conclude that it is the DPA taken up by the portal vein that liberates liver copper to cause serum and brain copper elevations. Consequently in WD patients, current clinical guidelines follow a “start low and go slow” strategy, that is, a careful upward titration of DPA over weeks and months to avoid a rapid transitory increase in toxic, non-ceruloplasmin–bound copper in the blood (62, 63).

As *Atp7b*^*−/−*^ rats shortly die upon hepatitis onset, we thus switched to cellular studies here to investigate potential toxicity to such copper pulses, that is, loosely albumin-bound copper. As the by far major copper binder in *Atp7b*^*−/−*^ rat serum is albumin, we thus used an albumin concentration close to the physiological range. Furthermore, using the reported affinity values for the diverse albumin copper-binding sites, we came up with the setting of different molar copper/albumin ratios to mimic situations with tightly versus more loosely bound copper. Clearly, these conditions are artificially high with respect to the added copper (in absolute amounts) here. It should, however, be mentioned that we investigated 24-h incubations (in contrast to years/decades of clinical silence in neurological WD). In fact, when a low ratio (1:1) was used, no cell toxicity in any of the tested cell types was encountered. This demonstrates the enormous binding capacity of albumin thereby avoiding acute cell toxicity. However, when the Cu to albumin ratio rose, cell viability went down, with endothelial cells being especially vulnerable. Importantly, an increased mitochondrial impairment and disruption of the cellular connectivity of endothelial cell layers resembling the first barrier of the BBB, appeared as early signs of such Cu–albumin toxicity, that is, without the initiation of cell death. Moreover, such an initial damage already allowed for progressive trans/para-epithelial copper passage. We therefore suggest endothelial BBB damage upon elevated loosely plasma protein-bound copper as one potential initial damage mechanism in neurologic WD that would subsequently allow for facilitated copper entry into the brain. It will be highly interesting to see in clinical settings whether this conceptual conclusion from our study can be validated.

Second, neurological worsening has been reported to occur frequently with DPA treatment but much less frequently upon TTM treatment. This could be due to a differentially altered presence of loosely plasma protein-bound copper upon diverse chelator treatment. The high-affinity copper chelator ALXN1840 caused the removal of loosely bound copper from albumin and its embedding into a deep cleft of the protein itself (termed tripartite complex TPC). Although it is unclear (due to the obtained low-resolution crystallography data) whether copper has also been removed from the high-affinity copper-binding site by ALXN1840, loosely bound copper was removed. Indeed, such TPC formation upon ALXN1840/TTM administration (either orally or injected) has been demonstrated in vivo in *Atp7b*^*−/−*^ rats and WD patients as well (35, 42, 64, 65). Consequently, hardly any signs of Cu–albumin toxicity were encountered in our study upon TPC formation. This may either be due to a lower cellular TPC uptake versus loosely bound copper resulting in lower cellular copper levels, or a firm continuous association of copper and ALXN1840 (even within cells in the studied time frames of 1–2 d). In contrast, DPA routed copper to the urine, demonstrating its passage into the blood and renal clearance. Such DPA-initiated copper urinal excretion was, however, quantitatively limited as only 10% of the net copper intake of *Atp7b*^*−/−*^ rats can be found in the urine upon DPA treatment (i.e., 0.4 μmol/24 h of a total net uptake of ∼3.95 μmol/24 h, unpublished observation). Nevertheless, renal copper clearance by DPA occurred fast (41), as 3 d after treatment stop, we did not observe elevated blood copper. As DPA is administered several times daily to WD patients, rapidly elevated blood copper peaks may arise. Such DPA-initiated copper peaks would be subjected to redistribution, positively to the urine but negatively to the brain. In fact, upon co-incubating different cell types with Cu–albumin and DPA, that is, avoiding an excretion route, these potentially negative effects became amply visible in our study. As with Cu–albumin alone, hardly any beneficial effect was encountered upon DPA presence. Thus, only when DPA-bound blood copper is excreted fast such negative effects may be avoided. Of note, another potential binding partner for DPA-mobilized liver copper may be the high-affinity binding site of albumin itself, potentially resulting in recirculating copper liver reuptake, thereby plausibly explaining why WD patient livers, even years after DPA treatment are still heavily burdened with copper (66).

And finally, neurological damage occurs frequently in WD patients. Given the early studies by Vogel et al (67, 68), who demonstrated direct copper toxicity in cat brains, but also other species, together with the strongly elevated copper levels in neurologic WD patients (10, 11, 12), the current consensus holds that copper is the prime responsible neurotoxin in these patients. Indeed, we found that all tested cell lines including surrogate neuronal and astrocytic cells, are highly vulnerable to copper that dissociates from albumin. As we and others have earlier reported that neurons have comparatively very low-protective metallothioneins (52, 69, 70), these cells appear relatively unprotected against copper insults. In this respect, copper-induced damage to the protective barrier cells, as demonstrated in our study, presents an enormous threat as it would cause a comparatively uncontrolled copper entry into the brain. In agreement, Stuerenburg suggested an involvement of the BBB in four neurologic WD patients showing neurological deterioration under DPA treatment paralleled by an increased BBB damage (27). As we did not observe a rescue of the endothelial BBB by DPA co-treatment, but DPA may cause massive copper mobilization into blood, this could have two detrimental consequences. First, especially neurologic WD patients with pre-damaged BBB would be highly vulnerable to such chelator-induced copper pulses, and second, elevated presence of such DPA-bound copper in blood could even worsen pre-existing BBB damage. This suggests that neurologic patients may be tested for BBB damage (e.g., by determination of S100B levels in blood or of the albumin ratio cerebrospinal fluid/serum) before DPA treatments are initiated. Importantly, we find that such damage or leakiness occurs already at non-toxic doses and identified endothelial cell mitochondria as one vulnerable target. In fact, Doll et al. described mitochondria as “key players in BBB permeability” (53). In agreement with our study, manipulation of mitochondrial respiration was paralleled with a rapid increase in BBB permeability and disruption of the tight junctions.

In summary, we propose the BBB as a highly sensitive structure to abrupt blood copper overload. In addition, we linked the occurrence of neurological worsening upon DPA co-treatment to its inability to rescue such damage. In contrast, high-affinity chelators seem to be much more protective in this respect. Indeed, ALXN1840 was found to effectively bind loosely attached albumin copper (in this case forming the tripartite complex), and largely avoided BBB copper toxicity. It will be interesting to extend the concept of this study in the future to further WD treatments, either already existing, that is, to zinc and trientine, or in development. Although we have admittedly used an *in vitro* system to demonstrate acute toxicity on the BBB constituting cells, this study nevertheless suggests such damage to be checked for in neurological WD patients. Given that earlier findings of such impairments in a few neurological WD cases hold true in more patients, our study suggests that BBB damage pre-screening should be envisioned before treatments with low/intermediate copper affinity chelators are initiated.

## Materials and Methods

### MicroPET/magnetic resonance imaging (MRI)

Wild-type rats underwent anatomical MRI 1T and dynamic PET (Mediso Medical Imaging Systems). Anesthesia with isoflurane was initiated with the rat placed in an acrylic glass chamber and maintained with respiration in a mask during the scan. A bolus of ^64^Cu (≈10 MBq/animal) was injected via a tail vein catheter or intraperitoneal. PET scanning was performed the first 120 min after injection, followed by a 25-min T1-weighted MRI scan. Body temperature and respiration frequency were monitored during anesthesia.

PET images were reconstructed with a three-dimensional ordered subset expectation algorithm (Tera-Tomo 3D; Mediso Medical Imaging Systems) with four iterations and six subsets and a voxel size of 0.6 × 0.6 × 0.6 mm^3^. Data were corrected for dead-time, decay, and randoms using delayed coincidence window without corrections for attenuation and scatter. The 120-min dynamic PET scans were reconstructed as 8 frames of 15 min and presented as standardized uptake value.

The animal study was approved by Dyreforsøgstilsynet under the Danish Ministry for Veterinary and Food Administration. The study was carried out in strict accordance with the recommendations in the Guide for the Care and Use of Laboratory Animals, EEC Council Directive 2010/63/EU.

### Animal studies

Animals were maintained under the Guidelines for the Care and Use of Laboratory Animals of the Helmholtz Center Munich. Animal experiments were approved by the government authorities of the Regierung von Oberbayern.

Control *Atp7b*^+/−^ and WD *Atp7b*^−/−^ rats were fed ad libitum with normal chow (1314; 13.89 mg Cu/kg; Altromin Spezialfutter GmbH) and tap water. All rats were healthy at treatment start and presented no signs of acute liver damage (serum aspartate aminotransferase <200 U/l and serum bilirubin <0.5 mg/dl). *Atp7b*^−/−^ rats (age: 79–96 d) were treated intraperitoneally for 4 consecutive days with 2.5 mg/kg body weight (bw) ALXN1840 once daily or 100 mg/kg bw DPA once daily. Untreated *Atp7b*^+/−^ and *Atp7b*^−/−^ rats served as controls. Urine and feces were collected at 24 h intervals for which rats were housed individually in metabolic cages for 4 d. After a 2 d resting period off treatment in normal cages and group housing, rats were euthanized for serum collection. Copper levels in urine, serum, and feces were analyzed by inductively coupled plasma optical emission spectrometry (ICP-OES, ARCOS, SPECTRO Analytical Instruments) as previously described (49).

### Gel filtration chromatography

10 mg of fatty acid–free bovine serum albumin (subsequently referred to as albumin, Carl Roth) was resuspended in 10 mM Tris–HCl buffer (pH 7.4) and mixed with 45 μl of 10 mM copper chloride. Where indicated, 45 μl of 10 mM ALXN1840 or DPA was added to the Cu–albumin complex before loading the samples onto a Superdex 75 10/300 GL column (GE Healthcare). 1 ml fractions were analyzed for protein content by the Bradford assay (71), molybdenum and copper levels by ICP-OES (49), and DPA content using 1,2-naphthoquinone-4-sulfonate (NQS) as previously described (72) with minor modifications. Briefly, 50 μl of each fraction was mixed with 10 μl 0.2% NQS, 10 μl of 0.2 M sodium phosphate buffer (pH 12.0), and 30 μl ddH_2_O in a clear 96-well plate. The samples were incubated for 20 min and absorbance was measured at 452 nm. Absolute levels of DPA were calculated using equally treated DPA standard solutions (25–250 μM).

### X-ray crystallography of the tripartite complex copper–albumin–ALXN1840 (TPC)

100 mg albumin was suspended in buffer containing 50 mM potassium phosphate and 150 mM sodium chloride (pH 7.5). Copper chloride and ALXN1840 were added in double molar excess and the mixture was incubated for 30 min at 37°C. The Cu–albumin–ALXN1840 mixture was loaded onto an S200 gel filtration column equilibrated with PBS (pH 7.4). Fractions corresponding to the Cu–albumin–ALXN1840 (TPC) in the monomeric state were pooled and protein was concentrated to 100 mg/ml. Screening for crystallization conditions was performed using commercially available buffer sets in a sitting-drop vapor-diffusion setup by mixing 0.2 μl of protein complex solution and 0.2 μl of buffer solution. Crystals were obtained at room temperature from a solution containing 0.1 M succinic acid, sodium dihydrogen phosphate, glycine buffer (pH 7.0), and 0.25% PEG 1500. Crystals were cryo-protected in 30% glycerol in the mother liquor and flash-cooled in liquid nitrogen. The diffraction data were collected at the ID23-2 beamline at the European Synchrotron Radiation Facility. The data were indexed and integrated using X-ray detector software (73, 74), scaled and merged using Scala (75). The initial phases were obtained by molecular replacement calculated using Phaser (76) and albumin structure as a search model (protein database 4F5S and reference 77). The initial model was manually rebuilt because of the resulting electron density maps using Coot (78). Because of the low resolution of data, refined structure did not reach R_free_ values below 0.40. Nevertheless, we were able to analyze a final model in terms of presence of ALXN1840 because of the high scattering factor of the molybdenum complex resulting in a strong detectable signal.

### EPR

For EPR measurements, complexes of Cu–albumin (2 mM/1 mM), Cu–albumin–ALXN1840 (TPC, 2 mM/1 mM/1 mM), and albumin–ALXN1840 (1 mM/1 mM) were prepared in 10 mM Tris/HCl (pH 7.4) buffer and reduced with an excess of sodium dithionite (Merck) shortly before measurements where indicated. EPR spectra were recorded at 77 K using an ECS106 spectrometer (Brucker BioSpin) operating in X-band at about 9.5 GHz.

### Cell culture

SHSY5Y (human neuroblastoma), U87MG (human astrocytoma), EA.hy926 (human endothelium), and HepG2 (human hepatocellular carcinoma) cells were from ATCC and were cultured in DMEM supplemented with 10% FCS (Biochrom) and 1% antibiotic-antimycotic (Life Technologies). All cells were maintained at 37°C in a humidified atmosphere with 5% CO_2_.

### Cell toxicity assays

2 × 10^4^ cells were seeded into each well of a 96-well plate and incubated overnight. On the next day, cells were treated for 24 h with increasing copper concentrations (0–2,500 μM) and 250 μM albumin (resulting in Cu–albumin molar ratios of 1:1, 2:1, 3:1, 4:1, and 10:1) in the absence or presence of 750 μM ALXN1840 or DPA in DMEM (2% FCS). Cell toxicity was either determined by CellTiter-Glo assay (Promega) or Trypan blue exclusion test (79).

### Cellular copper content

2 × 10^6^ cells were incubated for 24 h with 750 μM copper and 250 μM albumin (i.e., at a Cu–albumin molar ratio of 3:1) in the absence or presence of 750 μM ALXN1840 or DPA, respectively, in DMEM (2% FCS). Afterwards, cells were trypsinized and counted. Cell viability was determined by Trypan blue exclusion test. Copper and molybdenum content of cells was analyzed by ICP-OES (Ciros Vision, SPECTRO Analytical Instruments) as previously described (49).

### Electron microscopy

Electron microscopy of cells was performed as previously described (50) on a 1200EX electron microscope (JEOL) at 60 kv. Pictures were taken with a KeenView II digital camera (Olympus) and processed by the iTEM software package (analySIS FIVE, Olympus).

### Mitochondrial function

U87MG and EA.hy926 cells were pretreated for 24 h with DMEM (2% FCS) alone or with DMEM (2% FCS) containing 750 μM copper chloride and 250 μM albumin in the absence or presence of 750 μM ALXN1840 or DPA. Oxygen consumption was assessed by high-resolution respirometry using the Oxygraph-2k and DatLab 7.0 (Oroboros Instruments GmbH) as described previously (80). Per chamber, 1.5–2 × 10^6^ living cells were supplied in 2 ml of MiR05 buffer (0.5 mM EGTA, 3 mM MgCl_2_, 60 mM lactobionic acid, 20 mM taurine, 10 mM KH_2_PO_4_, 20 mM Hepes, 110 mM sucrose, 1 g/l albumin, pH 7.1) and routine respiration was measured. Addition of 2.5 μM oligomycin (inhibitor of the F_O_F_1_-ATPase) enabled the measurement of leak respiration and stepwise addition of CCCP (1 μl steps from 1 mM stock solution) allowed the determination of the maximum oxygen flux and thereby the capacity of the ETS. The oxygen flux was baseline-corrected for non-mitochondrial oxygen consuming processes (ROX) by the addition of 2.5 μM of the complex III–inhibitor antimycin A.

For complex IV activity measurements, cells were pretreated for 24 h with DMEM (2% FCS) alone or with DMEM (2% FCS) containing 750 μM copper chloride and 250 μM albumin in the absence or presence of 750 μM ALXN1840 or DPA. Complex IV activity was measured as previously described (81). Briefly, about 2.5 × 10^6^ cells were detached, washed two times with PBS by centrifugation and the cell pellet was resuspended in 200 μl of 20 mM hypotonic potassium buffer. After three freeze–thaw cycles, complex IV activity was measured by adding 10 μl of the sample to 90 μl of 50 mM potassium phosphate buffer (pH 7.0) containing 50 μM reduced cytochrome *c* with or without 0.3 mM KCN. Absorbance was measured at 550 nm for 10 min in a plate reader (Synergy 2, BioTek Instruments, Inc.) and complex IV activities were calculated from the linear slopes of the initial rates corrected for unspecific activity (in the presence of KCN) and normalized to the protein content determined by the Bradford assay (71).

### Endothelial BBB model

For transepithelial resistance (TEER) experiments (82), cryopreserved primary porcine brain capillary endothelial cells (PBCECs) were thawed and seeded either on rat tail collagen-coated 96-well plates for cytotoxicity testing using the neutral red assay (83) or Transwell inserts (area: 1.12 cm^2^, pore size: 0.4 μm; Corning) for barrier integrity studies. Cells were cultured for 48 h in Earle’s Medium 199 supplemented with 10% FCS, 50 U penicillin/ml, 50 μg/ml streptomycin, 100 μg/ml gentamycin, and 0.7 mM l-glutamine and maintained at 37°C in a humidified atmosphere with 5% CO_2_. Subsequently, the medium was changed to DMEM/Ham’s F 12 (1:1) containing 50 U penicillin/ml, 50 μg/ml streptomycin, 100 μg/ml gentamycin, and 4.1 mM L-glutamine and 550 nM hydrocortisone for additional 48 h upon which the medium was changed to the treatment solution containing 250 μM copper (and 83.3 μM albumin, Cu–albumin molar ratio 3:1) in the absence or presence of 250 μM DPA or ALXN1840, respectively. TEER and capacitance values were continuously monitored over 48 h using a CellZscope device (nanoAnalytics). Only PBCEC monolayers with initial TEER values > 600 Ω × cm^2^ and capacitance values between 0.45 and 0.6 μF/cm^2^ were used for permeability studies (84). The barrier integrity was calculated by normalizing the TEER values to the respective start values.

At treatment start, after ∼24 and 48 h exposure to the test substances, 20 μl of the apical medium as well as 40 μl of the basolateral medium were collected for subsequent copper determination. Total copper was determined by ICP-MS/MS as previously described (85).

For the immunocytochemical staining of tight junction proteins, confluent PBCECs cultivated on Transwell membrane inserts were processed as previously described (55). Briefly, PBCECs were fixed with formaldehyde and permeabilized using Triton X-100. After blocking of unspecific binding sites by albumin, the cells were incubated with the either anti–Claudin-5 or anti-ZO-1 antibody (Zytomed Systems GmbH). After a second blocking step, the cells were treated with an Alexa Fluor 488–conjugated secondary antibody (Invitrogen, Molecular Probes Inc.). Hoechst 33258 (Merck) was used to stain cell nuclei. Subsequently, membranes were cut out of the inserts and mounted in Aqua Poly/Mount (Polysciences Inc.). After a solidification period of 24 h, the samples were evaluated using a DM6 B fluorescence microscope by Leica Microsystems CMS GmbH in combination with the Leica Application Suite X.

Electron microscopy of PBCECs grown on Transwell inserts was performed as previously described (86) with minor modifications. Briefly, after fixation with 2.5% glutaraldehyde, cell monolayers were post-fixed with 1% osmium tetroxide for 30 min and dehydrated by ethanol. Cell monolayers were gradually embedded in epoxy resin in ethanol (1:2, 1:1, 2:1 for 20 min each) and finally embedded in 100% epoxy resin for 48 h at 60°C without pre-embedding before cutting and image acquisition.

### Miscellaneous/statistics

Chemicals were obtained from Sigma-Aldrich if not stated otherwise. DPA was a kind gift from Heyl Pharma. ALXN1840 was a kind gift from Alexion AstraZeneca Rare Disease.

Cellular protein levels were determined by the BCA assay (87). Cell size was determined using a LUNA-II Automated Cell Counter (Logos biosystems).

Throughout this manuscript “N” designates the number of biological replicates and “n” the number of technical replicates. Data are mean values with SD. Statistical significance was analyzed with the respective tests indicated in the figure legends using GraphPad Prism 7 (GraphPad Software Inc.).

## Supplementary Material

Reviewer comments
